# Correlation between high-risk Human Papillomavirus (HPV) viral load and cervical lesions across different subtypes, along with the development of a clinical prediction model

**DOI:** 10.1186/s12905-026-04707-5

**Published:** 2026-08-04

**Authors:** Hongyi Chen, Chenxi Yang, Hongwei Wang

**Affiliations:** 1 Graduate Training Base of Jinzhou Medical University at the Fourth Medical Center of the Chinese People’s Liberation Army General Hospital, 51 Fucheng Road, Haidian District, Beijing, 100037 China; 2 Department of Spine, Hand and Foot Surgery, The People’s Hospital of Yuhuan, Yuhuan, Zhejiang 317600 China; 3 Department of Pathology, The Fourth Medical Center of the Chinese People’s Liberation Army General Hospital, 51 Fucheng Road, Haidian District, Beijing, 100037 China

**Keywords:** HPV, Viral load, Cervical lesions, Nomogram, Subtype, Age

## Abstract

**Objective:**

The study aims to investigate the correlation between the viral loads of HPV16/18 and 16 other high-risk HPV types (designated as non-16/18 group) and the severity of cervical lesions, and to develop a HSIL+ risk prediction model to furnish evidence for pragmatic clinical triage and precision management strategies.

**Methods:**

A retrospective analysis was performed on the data from 331 HR-HPV-positive patients collected between July 2025 and January 2026. Using a semi-quantitative hybrid capture-chemiluminescence platform,The viral loads of HPV16/18 and non-16/18 group were measured, and patients were categorized into normal, LSIL, HSIL, and cervical cancer groups based on pathological findings.

Multivariate logistic regression analysis was employed to examine the associations among viral load, HPV subtypes, age, and lesion severity, and a HSIL+ prediction nomogram was constructed.

**Results:**

The prevalence of HPV16/18 infection escalated with increasing lesion severity (HSIL: OR=6.50; cervical cancer: OR=23.28), demonstrating a pattern of “continuous augmentation”. Conversely, the viral load of non-16/18 group increased during the LSIL and HSIL stages but declined in the cancer stage, exhibiting a pattern of “early involvement followed by later attenuation”. While single non-16/18 group infections demonstrated early warning value, HPV16/18 types and medium to high viral loads were identified as independent risk factors for HSIL+. The nomogram underwent Bootstrap validation and yielded an AUC of 0.763, indicating satisfactory calibration.

**Conclusion:**

This study comprehensively analyzed the pathogenic features of viral loads associated with HPV16/18 and other high-risk subtypes, elucidating the lesion patterns of distinct subtypes. While HPV16/18 acts as the principal persistent driver, the high viral load of the non-16/18 group provides a critical "window-of-opportunity" indication during the precancerous phase. A pragmatic HSIL+ risk-stratification nomogram was devised to serve as an accessible clinical aid for the early identification of high-risk lesions, thereby optimizing real-world personalized management.

**Supplementary Information:**

The online version contains supplementary material available at https://doi.org/10.1186/s12905-026-04707-5.

## Background

Cervical cancer is the fourth most common cancer among women in the world, and its incidence continues to rise [1]. In 2022, there were about 662,000 new cases and 349,000 deaths worldwide, among which the incidence rate was higher in low-income and middle-income countries [2, 3]. Continuous infection with high-risk human papillomavirus (HR-HPV) is the main cause of cervical cancer and precancerous lesions, which is associated with more than 95% of cervical cancer cases. Cervical intraepithelial lesions are divided into low-grade squamous intraepithelial lesions (LSIL) and high-grade squamous intraepithelial lesions (HSIL). Most LSIL cases can subside spontaneously, while untreated HSIL has a 30% risk of progressing to invasive cancer within 30 years [4]. Clinically, there is an urgent need for effective biomarkers to identify high-risk groups in the large population of HPV-positive patients, so as to achieve accurate risk stratification. Research shows that HR-HPV viral load may have predictive value [5]. However, its correlation with the severity of the lesion is still controversial [6–8], and little attention has been paid to subtypes except HPV16/18. Furthermore, in routine clinical screening, women testing positive for non-16/18 high-risk types often face significant triage dilemmas, as aggressive direct colposcopy may cause over-management, while simple delayed follow-up might miss concealed lesions. Understanding the nuanced subtype-specific viral load characteristics of these remaining 16 high-risk subtypes could provide an essential clinical window for risk stratification.To bridge this gap and elevate traditional correlation analysis into a pragmatic clinical tool, this study aims to explore the relationship between the viral loads of HPV16/18 and 16 other high-risk subtypes and the severity of cervical lesions, and to subsequently develop a localized, visualized risk prediction nomogram combining these platform-specific viral loads with host age, thereby providing a quantifiable and individualized basis for cervical cancer screening and risk stratification.

## Materials and methods

### Study population

A retrospective analysis was conducted on clinical data of HR-HPV-positive patients who underwent secondary screening and colposcopy at Yuhuan People’s Hospital from July 2025 to January 2026. Inclusion criteria: age 21–65 years old; tested for high-risk HPV DNA load by hybrid capture-chemiluminescence method and confirmed HR-HPV infection. Exclusion criteria [9–11]: pregnant or lactating patients; those who used antibiotics or hormones within 1 week; those who had sexual intercourse or vaginal douching within 3 days; patients with acute cervicitis or pelvic inflammatory disease; immunodeficiency diseases; history of hysterectomy, personal history of cervical cancer, or history of other malignant tumors.

### Sample collection

HPV specimen collection: The patient was instructed to assume the lithotomy position.

A professional gynecologist exposed the cervix using a disposable sterile speculum, inserted a specialized cervical brush into the cervical os, and rotated it clockwise 3 to 5 times to obtain an adequate number of exfoliated cervical cells. The brush head containing the cells was placed into a sampling tube containing cell preservation solution and sent for testing.All clinical specimens and colposcopic procedures were consistently performed by a dedicated team of senior gynecologists with over 5 years of colposcopy experience adhering to standardized clinical protocols to limit operator-dependent variations.

### HR-HPV viral load detection

This study used the BH8 Human Papillomavirus Nucleic Acid Detection Kit (Hangzhou Baiyin Biotechnology Co., Ltd.) to detect HPV16, HPV18, and 16 other high-risk HPV types (hereinafter uniformly referred to as non-16/18 group), including HPV26, 31, 33, 35, 39, 45, 51, 52, 53, 56, 58, 59, 66, 68, 73, 82) using the hybrid capture-chemiluminescence method. The assay employed a standardized commercial clinical kit, with built-in positive controls (pre-quantified HPV DNA plasmids) and internal quality controls to ensure technical validity.Viral load was expressed as the ratio of the relative light unit of the test sample to the cutoff value of the HPV DNA positive control (RLU/CO). RLU/CO ≥ 1 was considered positive, with 1–10 as low load, > 10–100 as medium load, and > 100 as high load [2]. Mixed infections were classified as HPV16/18 positive for statistical analysis.

### Pathological examination

A professional gynecologist performed an acetic acid test and iodine staining under colposcopy to identify suspicious lesion areas. For patients without suspicious lesions, random multi-point biopsies were taken at the 3, 6, 9, and 12 o’clock positions of the cervix in accordance with international colposcopy consensus guidelines to prevent topographically hidden high-grade lesions from being missed. According to the 5th edition of the WHO Classification of Female Genital Tumors, the pathological results were classified into four levels: within normal range as normal (WNL), low-grade squamous intraepithelial lesion (LSIL), high-grade squamous intraepithelial lesion (HSIL), and cervical cancer (CC). The highest grade lesion was taken as the final diagnosis, which was reviewed and confirmed by two pathologists.

### Observation indicators

Data on patient age, HR-HPV subtype and viral load, and cervical biopsy pathology results were collected. Patients were divided into a normal group (N group), an LSIL group, an HSIL group, and a CC group. The correlation of age, HPV16/18 type, and non-16/18 group load levels with lesion severity was analyzed, and the influencing factors for the severity of cervical lesions were explored.

### Statistical methods

SPSS 26.0 was used for statistical analysis. Measurement data: normally distributed data were expressed as ($\:\stackrel{-}{\mathrm{x}}\pm\:\mathrm{s}$), and comparisons among groups were performed using one-way analysis of variance; non-normally distributed data were expressed as [M(Q1,Q3)], and comparisons among groups were performed using the Kruskal-Wallis test. Count data were expressed as number of cases (%), and comparisons among groups were performed using the χ^2^ test or Fisher’s exact test. 

Bonferroni correction was applied for multiple comparisons. Multivariate unordered logistic regression was used to analyze the influencing factors for the severity of cervical lesions; binary logistic regression was used to screen for risk factors for HSIL+ and to construct a nomogram. The model performance was evaluated using the ROC curve (AUC), Bootstrap internal validation (*n*=1000), calibration curve, and Hosmer-Lemeshow test. *P*<0.05 was considered statistically significant.

## Results

### Patient characteristics

A total of 331 patients were included in this study, aged 21–65 years old, with a mean age of 44.16 ± 10.45 years old. Among them, there were 207 cases in the N group, 80 cases in the LSIL group, 28 cases in the HSIL group, and 16 cases in the CC group; 85 cases were HPV16/18 positive, and 246 cases were non-16/18 group positive; 157 cases had low viral load, 101 cases had medium viral load, and 73 cases had high viral load.

The difference in age distribution among groups with different lesion severities was statistically significant (*P* < 0.05), with the LSIL group having a lower age than the normal group (*P* < 0.05). The difference in HPV type distribution among groups with different lesion severities was statistically significant (*P* < 0.05): from LSIL to cervical cancer, the proportion of HPV16/18 infection showed an increasing trend (*P* < 0.05), while the proportion of non-16/18 group infection showed a decreasing trend (*P* < 0.05). See Table 1.

**Table 1:** Patient characteristics

Indicator	N (*n* = 207)	LSIL (*n* = 80)	HSIL (*n* = 28)	CC (*n* = 16)	χ^2^	*P*
Age [M(Q1,Q3)]	46.00(39.00,53.00)	41.00(31.25,50.00)	39.00(34.00,46.75)	44.00(36.75,52.25)	16.059	0.001
HPV16/18	43 (20.77)	15 (18.75)	14 (50.00)	13 (81.25)	33.891	<0.001
non-16/18 group	164 (79.23)	65 (81.25)	14 (50.00)	3 (18.75)		

### Viral load-lesion association across HR-HPV subtypes

#### Association for HPV16/18

The viral load of HPV16/18 showed a significant increasing trend with the worsening of lesion severity, with statistical significance (*P* < 0.05), and both the HSIL group and CC group had higher viral loads than the normal group (*P* < 0.05). Regarding viral load distribution, the difference among groups was statistically significant (*P* < 0.05): the proportion of low load in the HSIL group was lower than that in the N group, while the proportions of high load in the HSIL group and CC group were higher than that in the normal group (*P* < 0.05). The N group was predominantly low load, while the proportions of high load were significantly increased in the HSIL group and CC group. See Table 2.

**Table 2:** Correlation between HPV16/18 viral load and severity of cervical lesions

Indicator	N (*n* = 43)	LSIL (*n* = 15)	HSIL (*n* = 14)	CC (*n* = 13)	H/χ^2^	*P*
HPV16/18 viral load [M(Q1,Q3)]	2.70 (1.45,18.12)	10.81 (1.14,68.15)	38.07 (4.13,139.06)	37.11 (6.03,117.19)	12.954	0.005
Viral load distribution					14.308	0.018
Low load	30 (69.77)	7 (46.67)	4 (28.57)	6 (46.15)		
Medium load	10 (23.26)	6 (40.00)	5 (35.71)	2 (15.38)		
High load	3 (6.98)	2 (13.33)	5 (35.71)	5 (38.46)		

#### Association for non-16/18 group

The viral load of non-16/18 group was significantly higher in the LSIL group and HSIL group than in the normal group (*P* < 0.05). The viral load in the N group, LSIL group, and HSIL group increased with the rising grade of cervical lesions. Regarding viral load distribution, the difference among groups was statistically significant (*P* < 0.05): the proportions of low load in the LSIL group and HSIL group were lower than that in the N group, while the proportions of high load were higher than that in the N group (*P* < 0.05). The proportions of medium and high load in the LSIL group and HSIL group were significantly increased, showing an upward trend. See Table 3.

**Table 3:** Correlation between the viral load of non-16/18 group and the severity of cervical lesions

Indicator	N (*n* = 164)	LSIL (*n* = 65)	HSIL (*n* = 14)	H/χ^2^	*P*
non-16/18 group viral load [M(Q1,Q3)]	7.11 (2.02,42.18)	55.66(10.45,232.74)	59.38 (23.40,214.11)	34.468	< 0.001
Viral load distribution				43.098	< 0.001
Low load	94 (57.32)	14 (21.54)	0 (0.00)		
Medium load	45 (27.44)	24 (36.92)	8 (57.14)		
High load	25 (15.24)	27 (41.54)	6 (42.86)		

### Factors influencing the severity of cervical intraepithelial lesions

In this study, taking the severity of cervical intraepithelial lesions as the dependent variable, with the normal group as the reference, and taking patient age (with > 50 years old as the reference), viral load (with low load as the reference), and HPV type (with non-16/18 group as the reference) as independent variables, multivariate unordered logistic regression analysis was performed. The analysis results are shown in Table 4.

**Table 4:** Multivariate unordered logistic regression analysis of factors influencing the severity of cervical intraepithelial lesions

	Indicator	B	SE	Wald	*P*	OR	95%CI
LSIL	Intercept	-1.999	0.339	34.724	< 0.001		
	> 50 years old					1.000	
	20–30 years old	1.018	0.455	5.019	0.025	2.769	1.136–6.748
	31–40 years old	0.437	0.395	1.225	0.268	1.548	0.714–3.359
	41–50 years old	0.051	0.363	0.019	0.889	1.052	0.516–2.143
	non-16/18 group					1.000	
	HPV 16/18	0.106	0.362	0.086	0.769	1.112	0.547–2.262
	Low load					1.000	
	Medium load	1.082	0.335	10.437	0.001	2.952	1.531–5.693
	High load	1.695	0.368	21.243	< 0.001	5.449	2.650-11.205
HSIL	Intercept	-4.845	0.753	41.378	< 0.001		
	> 50 years old					1.000	
	20–30 years old	0.134	0.836	0.026	0.873	1.143	0.222–5.888
	31–40 years old	1.346	0.612	4.847	0.028	3.843	1.159–12.743
	41–50 years old	0.226	0.627	0.129	0.719	1.253	0.366–4.284
	non-16/18 group					1.000	
	HPV 16/18	1.871	0.476	15.490	< 0.001	6.498	2.559–16.501
	Low load					1.000	
	Medium load	2.346	0.632	13.797	< 0.001	10.443	3.028–36.009
	High load	2.921	0.675	18.716	< 0.001	18.569	4.943–69.757
CC	Intercept	-4.810	0.830	33.600	< 0.001		
	> 50 years old					1.000	
	20–30 years old	-0.658	1.242	0.281	0.596	0.518	0.045–5.907
	31–40 years old	0.007	0.848	0.000	0.993	1.007	0.191–5.303
	41–50 years old	0.423	0.674	0.395	0.530	1.527	0.408–5.719
	non-16/18 group					1.000	
	HPV 16/18	3.147	0.706	19.869	< 0.001	23.277	5.833–92.888
	Low load					1.000	
	Medium load	0.251	0.743	0.114	0.736	1.285	0.300-5.512
	High load	1.900	0.715	7.069	0.008	6.685	1.648–27.125

The study showed that, using the > 50 age group as the reference, the risk of LSIL was significantly increased only in the 20–30 years old age group (OR = 2.769), the risk of HSIL was significantly increased only in the 31–40 years old age group (OR = 3.843), and there was no statistically significant difference in risk across age groups for CC.

Viral load showed a clear dose-response relationship: compared with low load, the risk of LSIL for medium and high load was 2.952-fold and 5.449-fold, respectively; the risk of HSIL was 10.443-fold and 18.569-fold, respectively; for CC, only high load showed a significantly increased risk (OR = 6.685).Regarding HR-HPV subtypes, neither HPV16/18 nor non-16/18 group infection was significantly associated with LSIL; however, HPV16/18 significantly increased the risk of HSIL (OR = 6.498) and CC (OR = 23.277), and was the only strong independent risk factor for CC. To further delineate the clinical impact of the mixed infections within this high-risk cluster, a subgroup analysis was performed on the 85 HPV16/18-positive cases. Among them, 33 patients (38.82%) presented with mixed infections (co-infected with one or more non-16/18 group), exhibiting an HSIL+ prevalence of 42.42% (14/33). Conversely, 52 patients (61.18%) had pure single HPV16/18 infections, with an HSIL+ prevalence of 25.00% (13/52). A formal Pearson chi-square analysis demonstrated that although the observational rate of advanced lesions was notably higher in the mixed infection cluster, the correlation did not cross the strict threshold of statistical significance, exhibiting a marginal trend (x^2^= 2.802, *P* = 0.094). 

### Screening for risk factors of HSIL + and establishment of a clinical prediction model using binary logistic regression analysis

**Fig. 1:**
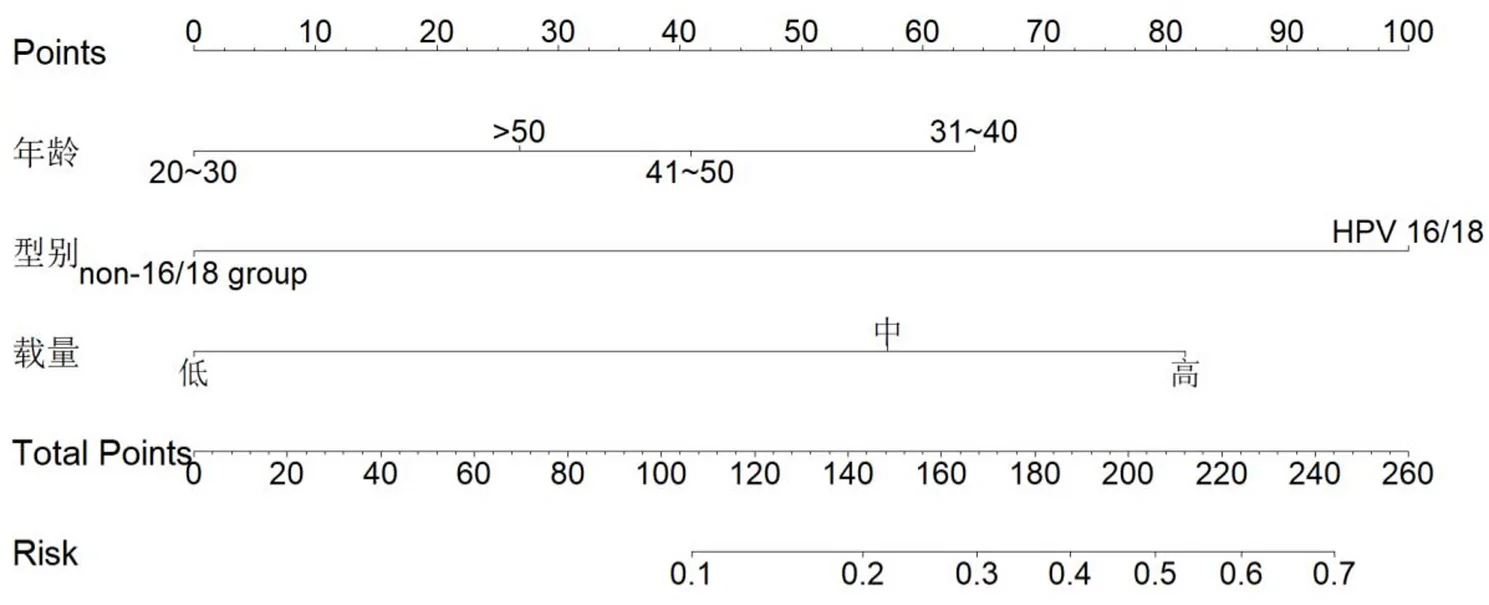
Nomogram of the clinical prediction model for HSIL+ based on predictors

Binary logistic regression analysis was performed with patient age, viral load, and HR-HPV type as independent variables and HSIL+ severity of cervical intraepithelial lesions as the dependent variable. The results indicated that HPV16/18 (OR = 9.167), medium load (OR = 3.545), and high load (OR = 6.102) were risk factors for HSIL + in patients (*P* < 0.05), as shown in Table 5. Based on the results of the multivariate binary logistic regression analysis, a visualized nomogram for predicting HSIL + in patients was constructed, as shown in Fig. 1.

**Table 5:** Results of multivariate logistic regression analysis for HSIL+

Indicator	B	SE	Wald	*P*	OR	95%CI
Intercept	-3.967	0.533	55.282	< 0.001		
> 50 years old					1.000	
20–30 years old	-0.594	0.701	0.717	0.397	0.552	0.140 ~ 2.182
31–40 years old	0.830	0.499	2.769	0.096	2.293	0.863 ~ 6.093
41–50 years old	0.313	0.477	0.431	0.511	1.368	0.537 ~ 3.485
non-16/18 group					1.000	
HPV 16/18	2.216	0.384	33.229	< 0.001	9.167	4.316 ~ 19.471
Low load					1.000	
Medium load	1.266	0.451	7.886	0.005	3.545	1.466 ~ 8.575
High load	1.809	0.485	13.883	< 0.001	6.102	2.357 ~ 15.800

### Validation of the clinical prediction model

**Fig. 2:**
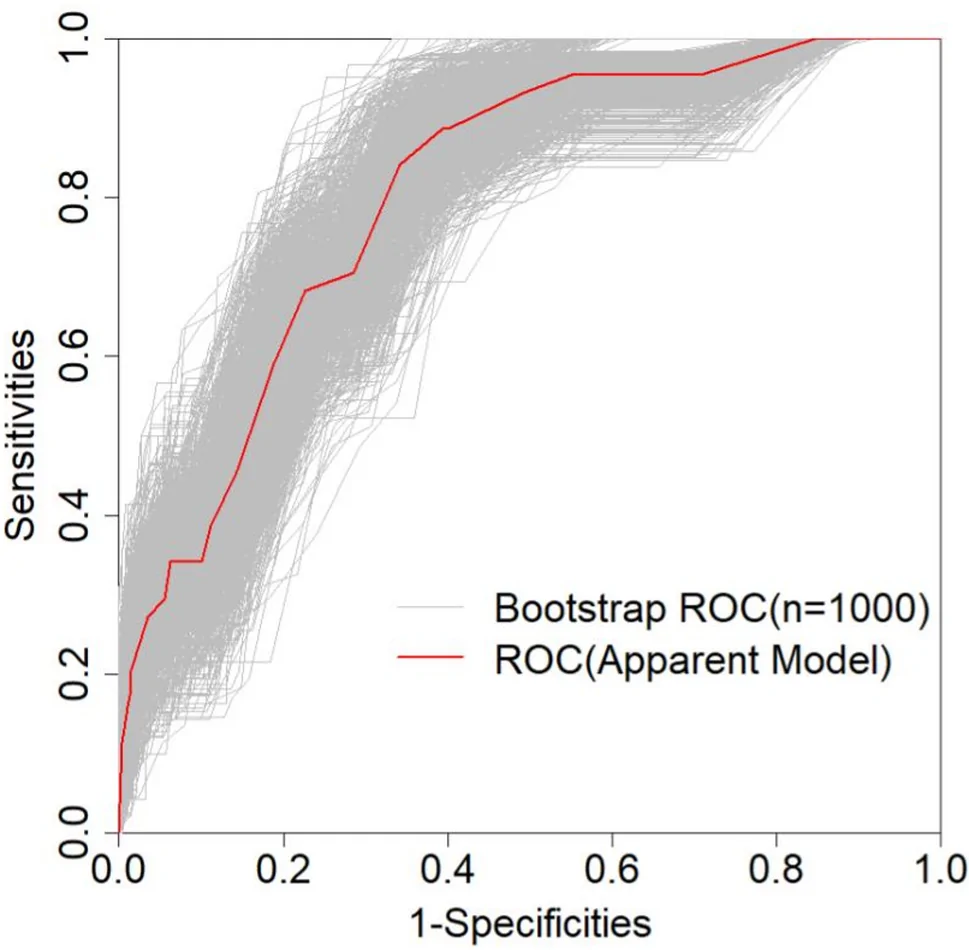
ROC curve and AUC of the clinical prediction model

The discriminative ability of this nomogram prediction model was evaluated using the ROC curve. The AUC of the ROC curve was 0.806 (95%CI: 0.743–0.869), with a sensitivity of 84.09% and a specificity of 65.85%. After Bootstrap resampling, the accuracy was 0.868, and the corrected AUC was 0.763 (95%CI: 0.641–0.853). The ROC curves of the datasets after Bootstrap sampling showed little fluctuation, indicating that the model has good discriminative ability, as shown in Fig. 2.

**Fig. 3:**
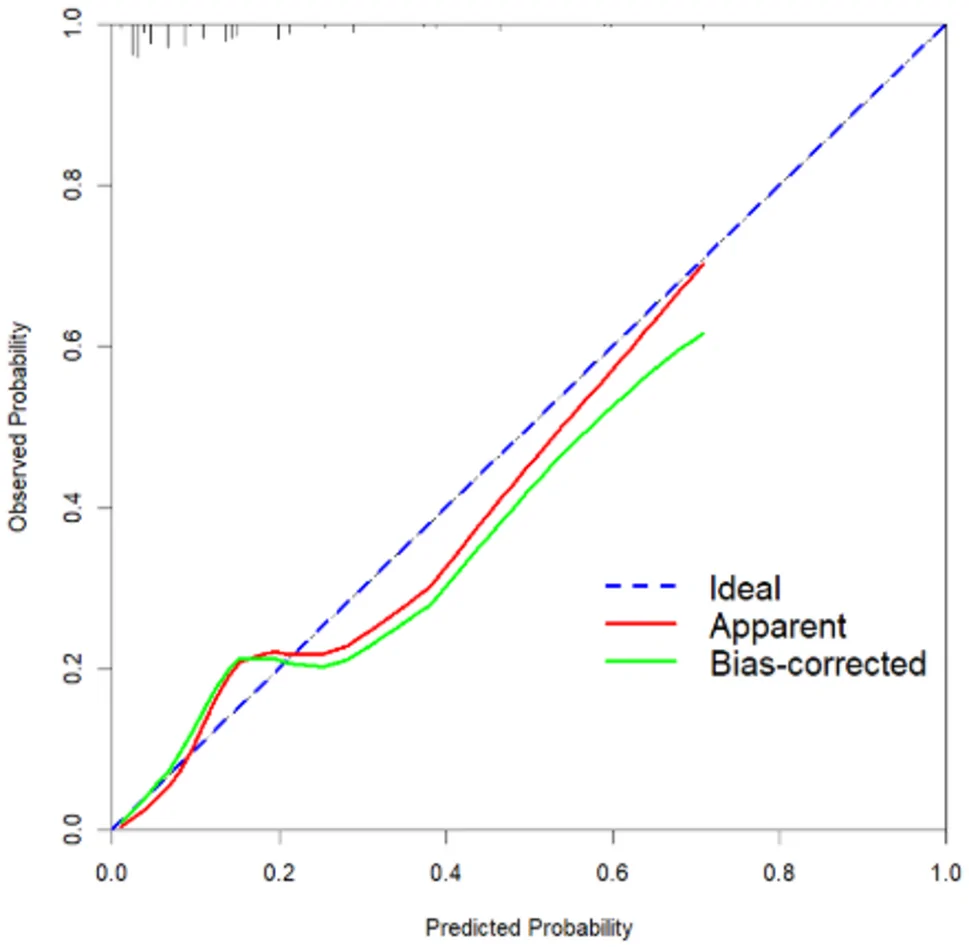
Calibration curve + H-L test of the clinical prediction model

The calibration of the prediction model was evaluated using a calibration curve and the Hosmer-Lemeshow goodness-of-fit test. Both the ideal curve and the calibration curve fitted well with the reference line, and the Hosmer-Lemeshow test (χ² = 11.283, *P* = 0.186 > 0.05) showed no statistically significant differences between the predicted results and the actual results, indicating that the prediction model has good predictive accuracy, as shown in Fig. 3. To ensure the robustness of our findings, a sensitivity analysis was performed by treating RLU/CO as a continuous variable via log_10_ transformation, which consistently yielded similar independent risk effects, confirming the excellent robustness of our model (Supplementary Table S1). 

## Discussion

In recent decades, the exploration of precise screening triage indicators for cervical cancer has continued to advance, among which HR-HPV viral load has been a focal point of controversy. Its significance in the detection and treatment of cervical lesions remains debated. This study used the hybrid capture-chemiluminescence method to detect the viral loads of HPV16/18 and non-16/18 group, systematically analyzing their cross-sectional correlation with the severity of cervical lesions and the influence of age. On this basis, a visualized nomogram for predicting HSIL + was further constructed, which provides a quantitative risk-stratification aid for clinical risk stratification.

This study finds that LSIL was most common in the 21–30 age group (22.50%), HSIL was most common in the 31–40 age group (42.86%), and and cervical cancer peaked in the 41–50 age group (50.00%). This shows a pattern of dynamic changes in the peak of lesions with age. The risk of harboring LSIL increased significantly in the 21–30 age group (OR = 2.769), but the proportion of cervical cancer was extremely low.

This phenomenon of “high infection, low carcinogenesis” is consistent with the HPV infection dynamics described by Lv et al. [12] Their study shows young women’s new infections accumulate rapidly and their viral loads are high, but their immune clearance capabilities are strong. Patel et al. [13] confirmed at the molecular level that cervical stromal fibroblasts in young women can significantly upregulate the expression of type I interferons and interferon-stimulated genes, thus enhancing antiviral ability. Therefore, for patients aged 21–30 with LSIL, excessive aggressive intervention should be avoided, and active monitoring should be the main strategy.

The risk of detecting HSIL in people aged 31–40 is significantly higher than that of people over 50 years old (OR = 3.843), suggesting that this age group is a high-risk period strongly associated with high-grade lesions. When the infection continues for more than 1–2 years, the risk of viral DNA integration increases, which may evade immune surveillance and be associated with advanced malignant changes [14]. The proportion of cervical cancer was highest in the age group of 41–50 years old, while the age group over 50 years old accounted for a considerable proportion of lesions at all stages (LSIL 23.75%, HSIL 17.86%, cervical cancer 25.00%). Qian Deying et al. [15] pointed out that the probability of identifying CIN2 + lesions in individuals over 50 years old with high HR-HPV load is as high as 96.0%. It is worth noting that regression analysis showed that after entering the cervical cancer stage, the risk difference among each age group was not statistically significant (OR values were close to 1). This indicates that once carcinogenesis occurs, age itself is no longer the main influencing factor. In clinical practice, attention should be paid to the history of HPV infection and the duration of continuous infection. Even when the viral load is low, more aggressive screening strategies and higher screening frequency should be adopted to facilitate early detection of potential precancerous lesions and cancerous changes.Reflecting these clinical realities, although the individual age brackets did not reach statistical significance (*P* > 0.05) in our multivariable model, Age was intentionally retained as a scoring parameter in the predictive nomogram.

Methodological guidelines for clinical prediction models (e.g., Steyerberg [16] et al.) suggest that well-established biological predictors should be retained in the final model regardless of their statistical significance in a specific small dataset to preserve clinical relevance. In our cohort, ample epidemiological evidence confirms that age remains inextricably linked to persistent HR-HPV duration and lesion severity [13, 15] Excluding age solely based on data-driven P-values would compromise the model’s clinical utility and generalizability in real-world risk stratification.

Regarding the differential pathogenic characteristics of HPV16/18 and non-16/18 group, the study found that from LSIL to cervical cancer, the proportion of HPV16/18 infection significantly increased (χ²=33.891, *P* < 0.001); while the opposite trend was observed for non-16/18 group. The pathogenicity of HPV16/18 showed “stage-dependent enhancement”: there was no significant association with HPV subtype risk at the LSIL stage, the risk increased to 6.498-fold at the HSIL stage, and reached 23.277-fold at the cervical cancer stage. This is consistent with the highest carcinogenic risk of HPV16/18 indicated by the IARC [17]. The viral load of HPV16/18 was high in both the HSIL and cervical cancer groups, further supporting its value for risk stratification in precancerous lesions and carcinogenesis. In contrast to the “continuous augmentation” pattern of HPV16/18, the pathogenic characteristics of non-16/18 group in cervical lesions showed a pattern of “early involvement followed by later attenuation”. This distinct trajectory represents a major highlight of our study, distinguishing it from traditional research that exclusively focuses on HPV16/18. The viral load of non-16/18 group was significantly higher in the LSIL and HSIL groups than in the normal group, and the proportion of medium and high load increased stepwise with lesion grade (normal group 57.32% → LSIL group 78.46% → HSIL group 100%, χ²=43.098, *P* < 0.001), suggesting its auxiliary triage value. Notably, this striking 100% high-load rate during the precancerous stages (LSIL/HSIL) underscores that a phase-specific heavy burden holds critical “window-of-opportunity” indicator value for clinical surveillance, catching potentially progressive cases before late-stage attenuation occurs.However, the proportion of non-16/18 group infection significantly decreased at the cervical cancer stage, and its carcinogenic efficacy was weaker than that of HPV16/18. This late-stage attenuation is likely driven by the molecular mechanism of viral integration into the host genome, which frequently disrupts the L1 or E2 open reading frames and consequently diminishes active viral replication [18].Some literature reports that no graded correlation was found between the viral load of the other 12 h-HPV types and cervical lesions [19]. This may be attributable to differences in study populations, subtype heterogeneity, and detection methods. A study from Yuebei People’s Hospital confirmed that for each log unit increase in the load of HPV52, 58, and 68, the risk of CIN1 + increased by 31%, 37%, and 49%, respectively [20]; Zhou et al. found that the loads of HPV31, 33, 35, 52, and 58 were significantly correlated with lesion grade [21]. Therefore, for patients who are HPV16/18 negative but have high loads of other high-risk types, simple neglect is not appropriate; their unique “early-peak” risk profile demands that close follow-up should still be conducted in conjunction with load levels to secure timely clinical intervention.Beyond this broad subtype stratification, we further explored the internal heterogeneity within the HPV16/18-positive stratum. Although the prevalence of HSIL + was observationally higher in patients with mixed infections (HPV16/18 combined with non-16/18 group) compared to those with pure single infections (42.42% vs. 25.00%), the difference was statistically marginal (x^2^ = 2.802, *P* = 0.094).This lack of statistical significance stems primarily from the limited sample sizes within these specialized subgroups, which inevitably compromises the statistical power to detect moderate clinical differences. Biologically, however, this explicit gap strongly implies a synergistic oncogenic trend, suggesting that co-infecting strains may cooperatively evade mucosal immune surveillance.Clinically, while pooling these mixed cases into a unified category streamlined our predictive nomogram, clinicians should remain vigilant regarding the heightened risk profile of such cumulative viral profiles during real-world screening and triage.

This study further used multivariate binary logistic regression analysis to identify HPV16/18, medium load, and high load as independent risk factors for HSIL+, and based on this, a nomogram was constructed to enable personalized decision-making regarding lesion risk. Clinicians can calculate the total score according to the assigned values of each risk factor subgroup in the nomogram, so as to obtain an individualized predicted probability of harboring HSIL+. This helps to better identify high-risk patients and optimize screening efficiency. After the model was established, the ROC curve and the calibration curve were used for validation. The results showed that the apparent AUC of the model is 0.806. After internal validation and correction of Bootstrap, the AUC was 0.763, indicating that the model has good discriminative ability and no obvious overfitting phenomenon. The calibration curve also showed that the prediction model has good prediction accuracy. Compared with single correlation analysis, the model integrates previously dispersed information into a single risk-stratification aid, enabling clinicians to quickly identify high-risk patients and optimize screening triage decisions. It is of great practical significance for the cervical screening management in resource-limited areas.

Several limitations of this study should be acknowledged. First, our localized 331-patient cohort features an uneven group distribution with a restricted sample size of advanced lesions (n = 16) and mixed infections (n = 85). Due to this scarcity of positive events, when expanding dummy variables for multivariable analysis, the parameter-based Events Per Variable ratio (EPV ≈ 7.3) falls below the recommended threshold. This limitation introduces statistical instability that widened the 95% confidence intervals for certain effect sizes and caused the localized calibration deviation at higher predicted probabilities (> 0.6) in Fig. 3, while also restricting our statistical power to fully evaluate the sub-stratum synergistic effects of mixed infections.Nevertheless, the predictor-based EPV stands at an adequate 14.67, and our 1,000-replicate Bootstrap validation yielded a stable bias-corrected AUC of 0.763.

This confirms acceptable internal stability, rendering the nomogram justifiable strictly as a pragmatic screening aid rather than a final diagnostic tool.Second, pooling non-16/18 genotypes into a single ‘non-16/18 group’ category constitutes a trade-off dictated by the technical specifications of our commercial assay. While this masks individual subtype heterogeneity, it directly reflects the high-throughput screening realities of frontline care settings. Third, behavioral cofactors (e.g., smoking, parity) and host immune factors were unavailable, potentially introducing residual confounding. Biologically, factors like smoking and long-term contraceptive use can impair localized mucosal immunity, which could cooperatively be linked with viral persistence and severe cervical lesions alongside high viral loads. Consequently, the omission of these host and lifestyle variables might have partially over- or underestimated the independent effect sizes (OR values) plotted in our screening model. Fourth, given the retrospective and cross-sectional architecture of our cohort, this study captures a static epidemiological snapshot. These observed associations must therefore be interpreted cautiously as cross-sectional correlations rather than definitive evidence of longitudinal disease progression, precluding definitive causal establishment. Consequently, future large-scale, prospective, multi-center studies with robust external validation using larger cohorts and full-genotyping datasets are explicitly warranted to handle the limited sample size, mitigate overfitting risks, fully adjust for these confounding cofactors, and confirm the model’s generalizability.

## Conclusion

To conclude, this study systematically reveals the cross-sectional correlations among age, HR-HPV viral load, and subtypes with the severity of cervical lesions. The lesion peak changes dynamically with age, and age is a strong correlate in the precancerous stage, but its association disappears in the cancerous stage. HPV16/18 shows a pathogenic pattern of “continuous augmentation” and represents the predominant pragmatic triage indicator associated with cervical cancer. Crucially, non-16/18 group presents a pattern of “early involvement followed by later attenuation”, demonstrating that its phase-specific heavy viral load holds invaluable clinical warning value during the precancerous “window-of-opportunity”. On this basis, this study constructs a clinical risk prediction nomogram for HSIL+ specifically tailored to the semi-quantitative viral load characteristics and subtype stratification of the hybrid capture-chemiluminescence platform. After internal validation, it showed good discriminative ability and calibration (AUC = 0.806). While based on a semi-quantitative platform, this nomogram successfully translates traditional correlation analysis into a pragmatic risk-stratification aid, providing an accessible, visual, and quantifiable decision-support framework to optimize the individualized triage of HR-HPV-positive populations within current real-world clinical practices.

## Supplementary Information

Table S1:Sensitivity Analysis of the Multivariable Regression Model Using Continuous log10 Viral Load.

## Data Availability

Patient data were protected and confidential. The datasets presented in this article are not publicly available due to hospital policy. The datasets used and analyzed during the current study are available from the corresponding author upon reasonable request.

## Abbreviations

HR-HPV: High-risk human papillomavirus
LSIL: Low grade squamous intraepithelial lesions
HSIL: High grade squamous intraepithelial lesions
WNL: Within normal range
CC: Cervical cancer

## References

[1] Fobian SF, Mei X, Crezee J, et al. Increased human papillomavirus viral load is correlated to higher severity of cervical disease and poorer clinical outcome: a systematic review[J]. J Med Virol. 2024;96(6):e29741. 10.1002/jmv.29741. 38922964

[2] Chen Y, Huang Q, Hong M, et al. Application value of high-risk HPV viral load detection in cervical cancer screening and triage: a real-world retrospective study based on cervical cancer screening in Quanzhou City, Fujian Province[J]. Chin J Obstet Gynecol. 2025;60(03):193–201. 10.3760/cma.j.cn112141-20241210-00657. 40159044

[3] Chen X, Yang Z. Research progress on the correlation between HR-HPV load and cervical intraepithelial lesions and cervical cancer[J]. Int Med Health Guidance News. 2024;30(6):932–5. 10.3760/cma.j.issn.1007-1245.2024.06.010.

[4] Vellone V, GEvaluating. HPV viral load and multiple infections for enhanced cervical cancer risk-based assessment[J]. Life. 2025;15. 10.3390/life15020153. PMC11856100 40003564

[5] Josefsson AM, Magnusson PKE, Ylitalo N, Sørensen P, Qwarforth-Tubbin P, Andersen PK, et al. Viral load of human papilloma virus 16 as a determinant for development of cervical carcinoma in situ: a nested case-control study[J]. Lancet. 2000;355(9222):2189-93. 10.1016/S0140-6736(00)02401-6 10881891

[6] Liu Y, Xu C, Pan J, et al. Significance of the viral load of high-risk HPV in the diagnosis and prediction of cervical lesions: a retrospective study[J]. BMC Women’s Health. 2021;21(1):1–6. 10.1186/s12905-021-01493-0. PMC8501737 34625069

[7] Zou S, Wang H. Clinical analysis of high-risk human papillomavirus load and cervical lesions[J]. Maternal Child Health Care China. 2018;33(18):3. DOI:CNKI:SUN:ZFYB.0.2018-18-016.

[8] Andersson S, Safari H, Mints M, et al. Type distribution, viral load and integration status of high-risk human papillomaviruses in pre-stages of cervical cancer (CIN)[J]. Br J Cancer. 2005;92(12):2195. 10.1038/sj.bjc.6602648. PMC2361820 15942630

[9] Zhang M, Cao D, Li C. Vaginal microecological characteristics of patients with cervical CIN2 and their relationship with peripheral blood lymphocytes[J]. Chin J Family Plann. 2025;33(3):723–6.

[10] Ma X, Zhang Y, Zhang H, et al. Correlation analysis between HR-HPV load and cervical lesions[J]. Med J Chin People’s Liberation Army. 2012;37(5):5. DOI:CNKI:SUN:JFJY.0.2012-05-021.

[11] Shao Z, Qi X, Yang L, et al. Level and clinical significance of high-risk human papillomavirus load in patients with cervical intraepithelial neoplasia[J]. Chin J Nosocomiology. 2021;31(22):3467-71.

[12] Lv P, Zhao F, Xu X, et al. Correlation between Common Lower Genital Tract Microbes and High-Risk Human Papillomavirus Infection[J]. The Canadian journal of infectious diseases & medical microbiology = Journal canadien des maladies infectieuses et de la microbiologie medicale /. AMMI Can. 2019;2019(2):1–6. 10.1155/2019/9678104. PMC6893239 31885754

[13] Patel MV, Shen Z, Hopkins DC, et al. Aging selectively alters PRR and ISG expression in endo- and ecto‐cervical stromal fibroblasts from the human female reproductive tract[J]. Am J Reprod Immunol. 2025;93(1). 10.1111/aji.70031. PMC12108118 39777771

[14] Yang J, Xue F. Research progress of immune mechanisms related to persistent HPV infection in CIN after cervical conization[J]. Iran J Allergy Asthma Immunol. 2024;23(5). 10.18502/ijaai.v23i5.16755. 39586741

[15] Qian D, Huang H, Cen J, et al. Role of high-risk human papillomavirus load combined with age in the triage of atypical squamous cells of the cervix[J]. J Practical Med. 2008;24(13):2. 10.3969/j.issn.1006-5725.2008.13.036.

[16] Steyerberg EW. Clinical prediction models: a practical approach to development, validation, and update[M]. 2nd ed. Cham: Springer; 2019. 10.1007/978-3-030-16399-0.

[17] Weiderpass E. International agency for research on cancer[J]. Encyclopedia Toxicol. 2024;573–6. 10.1016/b978-0-12-824315-2.00046-4.

[18] Cancer Genome Atlas Research Network. Integrated genomic and molecular characterization of cervical cancer[J]. Nature. 2017;543(7645):378–84. 10.1038/nature21384. PMC5354998 28112728

[19] Wang M, Hou B, Wang X et al. Diagnostic value of high-risk human papillomavirus viral load on cervical lesion assessment and ASCUS triage[J]. Cancer Med, 2021;(2). 10.1002/cam4.3653. PMC7982632 33682355

[20] Zhou J, Ma B, Ji J, et al. HPV16/HPV58 viral load is non-linearly correlated with cervical lesions and can be used as a triage marker[J]. Infect Agents Cancer. 2025;20(1). 10.1186/s13027-025-00684-w. PMC12281751 40696417

[21] Zhou Y, Liu J, Chen S, et al. Correlation of different HPV genotype viral loads and cervical lesions: a retrospective analysis of 1585 cases[J]. Cancer Cytopathol. 2025;133(1). 10.1002/cncy.22920. PMC11695744 39555989

