# Supplementary material for: Correlation between high-risk Human Papillomavirus (HPV) viral load and cervical lesions across different subtypes, along with the development of a clinical prediction model
Source: BMC Womens Health. 2026 Aug 4;26:573. doi: 10.1186/s12905-026-04707-5 (PMC13721635; doi:10.1186/s12905-026-04707-5)
Supplement: Supplementary file 1 [file 12905_2026_4707_MOESM1_ESM.pdf]

Table S1. Sensitivity Analysis of the Multivariable Regression Model Using Continuous log10 Viral Load.

Indicator	B	SE	Wald	*P*	OR	95%CI
Intercept	-4.154	0.558	55.347	<0.001		
>50 years old					1.000	
20-30 years old	-0.456	0.690	0.436	0.509	0.634	0.164~2.452
31-40 years old	0.854	0.496	2.968	0.085	2.348	0.889~6.202
41-50 years old	0.289	0.476	0.370	0.543	1.335	0.526~18.978
Type B					1.000	
HPV 16/18	2.195	0.382	33.105	<0.001	8.984	4.253~18.978
log10 Viral Load	0.859	0.229	14.114	<0.001	2.360	1.508~3.693
